# Critical Current and Pinning Features of a CaKFe_4_As_4_ Polycrystalline Sample

**DOI:** 10.3390/ma14216611

**Published:** 2021-11-03

**Authors:** Armando Galluzzi, Antonio Leo, Andrea Masi, Francesca Varsano, Angela Nigro, Gaia Grimaldi, Massimiliano Polichetti

**Affiliations:** 1 Department of Physics “E.R. Caianiello”, University of Salerno, Via Giovanni Paolo II 132, Fisciano, I-84084 Salerno, Italy; aleo@unisa.it (A.L.); anigro@unisa.it (A.N.); 2 CNR-SPIN Salerno, Via Giovanni Paolo II 132, Fisciano, I-84084 Salerno, Italy; gaia.grimaldi@spin.cnr.it; 3 ENEA, Via Anguillarese 301, I-00123 Roma, Italy; andrea.masi@enea.it (A.M.); francesca.varsano@enea.it (F.V.); 4 Dipartimento di Ingegneria, Università degli Studi Roma Tre, Via Vito Volterra 62, I-00146 Roma, Italy

**Keywords:** iron-based superconductors, 1144 IBS family, DC magnetic properties, pinning properties, pinning force analysis, magnetism and superconductivity

## Abstract

We analyze the magnetic behavior of a CaKFe4As4 polycrystalline sample fabricated by a mechanochemically assisted synthesis route. By means of DC magnetization (M) measurements as a function of the temperature (T) and DC magnetic field (H) we study its critical parameters and pinning features. The critical temperature T_c_ has been evaluated by M(T) curves performed in Zero Field Cooling-Field Cooling conditions. These curves show the presence of a little magnetic background for temperatures above T_c_, as also confirmed by the hysteresis loops M(H). Starting from the M(H) curves, the critical current density J_c_ of the sample has been calculated as a function of the field at different temperatures in the framework of the Bean critical state model. The J_c_(H) values are in line with the ones reported in the literature for this typology of samples. By analyzing the temperature dependence of the critical current density J_c_(T) at different magnetic fields, it has been found that the sample is characterized by a strong type pinning regime. This sample peculiarity can open perspectives for future improvement in the fabrication of this material.

## 1. Introduction

Until 2016, with the discovery of the 1144 Iron-Based superconductors (IBSs) family [1], the 122 and 11 IBS families have been the most studied due to their low anisotropy values, high values of critical current density J_c_, irreversibility field and upper critical field [2,3,4,5,6,7,8,9,10]. In this framework, the 1144 family [1] attracted significant interest in recent years due to the stoichiometric nature of its layered structure. This group of IBSs shares in fact key structural elements with the 122 family [11], well known for the optimum results obtained following their application in the production of superconducting wires [12]. The 1144 and 122 compounds are both characterized by the intercalation of mixed Alkaline (A) or Alkaline Earth (AE) ions between Fe-As planes, with the difference that in the 1144 structure A and AE elements do not mix in the same planes. The rigid alternation of A and AE elements gives rise to the formation of stoichiometric superconductors characterized by hole doping levels close to the optimal 0.4 observed in common 122 IBS, with critical temperatures above 35 K and high critical currents.

Regarding the superconducting critical currents, it has been observed in 1144 single crystals that the alternation of the elements in the intercalating planes can give rise to the formation of peculiar lattice defects that resemble stacking faults observed in cuprates [13,14,15]. The presence of these defects has been associated with the high critical currents observed in 1144 single crystals and with peculiar second magnetization peak phenomena observed in such crystals, and linked to the material pinning dynamics. In this work, we have analyzed the critical current density as a function of field and temperature together with the pinning features of a polycrystalline sample. Specifically, after having obtained the T_c_ of the sample, we have extracted the J_c_(H) curves starting from the M(H) loops comparing their values with the literature. After that, by analyzing the J_c_(T) curves’ behavior and the pinning force density as a function of the reduced field F_p_(h) curves, a strong pinning regime has been identified. The obtained results show that the sample can be considered for high-power applications due to its strong pinning behavior and to the possibility to enhance the critical current density values by improving the fabrication processes.

## 2. Materials and Methods

We have analyzed a disk-shaped pellet with diameter and thickness equal to 3 mm and 0.65 mm, respectively. The polycrystalline sample was obtained via a mechanochemically assisted synthesis route [16]. Briefly, elemental powders were subjected to a milling treatment using a steel vial and steel balls in a 30:1 ball to powder mass ratio for 5 h. The pressed powders were then subjected to thermal treatment for 10 h at 750 °C, adopting a 10 °C/min heating rate and a 5 °C/min cooling rate. With respect to our previous work, a slight imbalance in the starting Ca:K ratio was introduced in order to minimize K-122 phase segregation [17] starting thus from a nominal Ca:K:Fe:As = 1.2:1.18:3.75:4 atomic ratio. XRD diffraction patterns (data not shown) show the distinctive peaks of the p4/mmm 1144 phase [1] as in our previous work [17] and do not highlight significant presence of secondary phases (only traces of K-122 and CaO are evident). The sample has been characterized by means of DC magnetic measurements applied perpendicularly to the disk surface. The temperature and field dependence of the DC magnetization M(T) and M(H), respectively, has been measured by means of a QD PPMS doted of a VSM insert. To avoid the effect on the sample response due to the residual trapped field inside the PPMS DC magnet [18], this field was reduced below 1 × 10^−4^ T [19]. The sample has been cooled down to 2.5 K in zero field, then the H has been applied and the data have been gained for rising temperatures (Zero Field Cooling) up to 300 K. Then, the sample has been cooled again during the acquisition of the Field Cooling magnetization. For what concerns the M(H) measurements, the sample has been cooled to the interested temperature in zero field. Then, H was ramped up to +9 T, down to −9 T, and to +9 T again for acquiring the complete M(H) loops [20].

## 3. Results and Discussion

**Figure 1:**
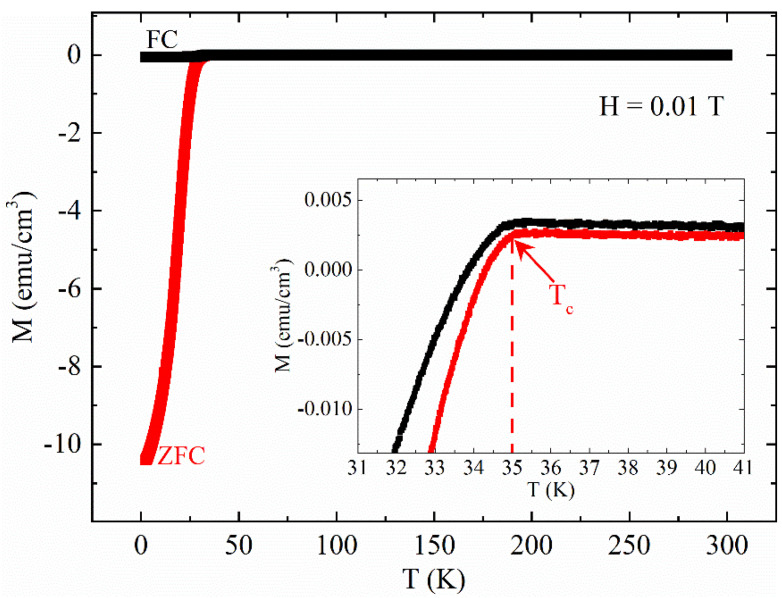
Magnetization versus temperature M(T) measured in ZFC-FC conditions at H = 0.01 T. Inset: T_c_ is indicated by a red arrow.

To obtain the T_c_ of the sample, a M(T) measurement has been made in Zero Field Cooling (ZFC)-Field Cooling (FC) conditions by using 0.01 T. The M(T) at H = 0.01 T is reported in Figure 1. The T_c_ has been individuated as the beginning of the ZFC M(T) transition (see inset of Figure 1). This value is approximately 35 K and it is consistent with the values reported in the literature [1,21,22]. It is worth noting the presence of a slight non-zero signal above T_c_ in the ZFC curve as already reported in other works on iron-based systems [23,24,25,26], which can be limited by improving the fabrication processes [26,27,28].

**Figure 2:**
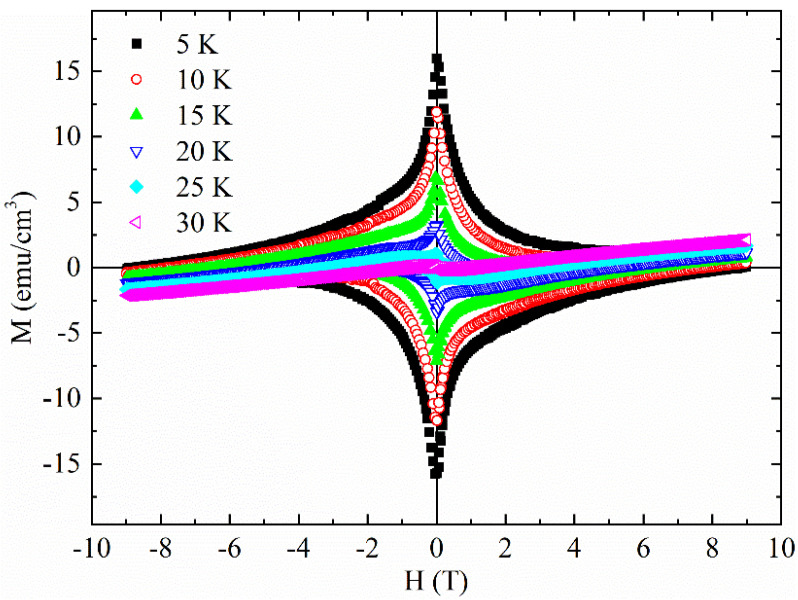
Magnetization versus field at different temperatures below T_c_.

The M(H) measurements have been performed at different temperatures in the range between 5 K and 40 K (see Figure 2). The decrease of the hysteresis areas with increasing temperature and the shape of M(H) loops imply the existence of flux pinning centers. Moreover, the not perfect symmetry of the superconducting hysteresis loops indicates the possible presence of surface barriers [29,30].

**Figure 3:**
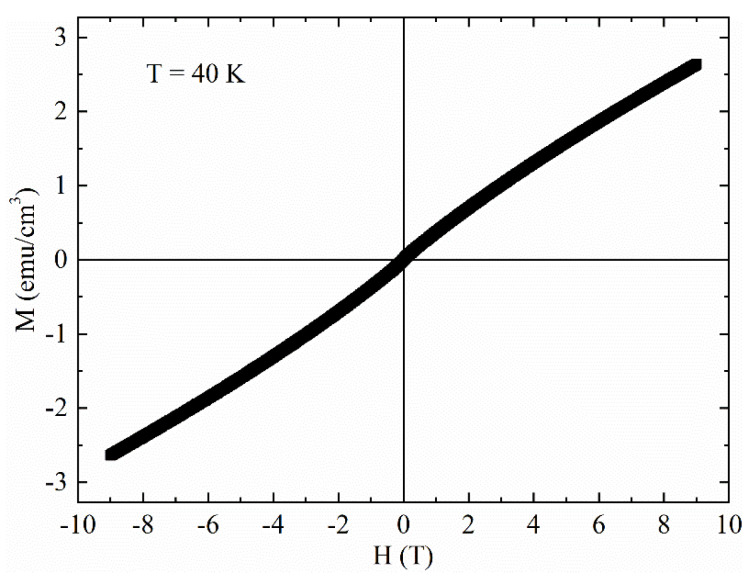
Magnetic hysteresis loop at T = 40 K.

It is visible a slight tilt of the curves probably due to the presence of a magnetic background or to magnetic impurities already detected in ZFC curve of Figure 1. In this framework, Figure 3 shows the M(H) curve at T = 40 K. At this temperature, the sample is in the normal state, so approximately this curve represents the magnetic background of the sample, which coexists with superconducting signal at lower temperatures T < T_c_. In particular, the coercive field is ≈100 Oe.

**Figure 4:**
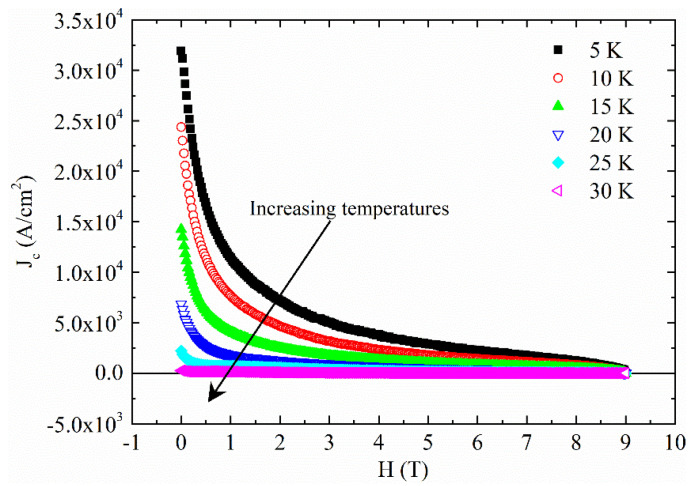
J_c_ versus field at different temperatures.

To analyze the transport and pinning properties of the sample, the critical current densities as a function of the magnetic field J_c_(H) and the temperature J_c_(T) have been studied. In particular, the J_c_(H) at different temperatures have been calculated by means of the formula [31,32]:

(1) $$J_{c}=\frac{30\Delta M}{d},$$

where $\Delta M=M_{\mathrm{dn}}-M_{\mathrm{up}}$ is the difference between the magnetization measured for decreasing (M_dn_) and increasing (M_up_) applied field, respectively, and *d* is the diameter. The ΔM is expressed in emu/cm^3^. In Figure 4, the obtained J_c_(H) curves have been reported for different temperatures. The obtained J_c_ values are in agreement with the ones reported in the literature for other polycrystalline CaKFe_4_As_4_ samples [16,22,33].

**Figure 5:**
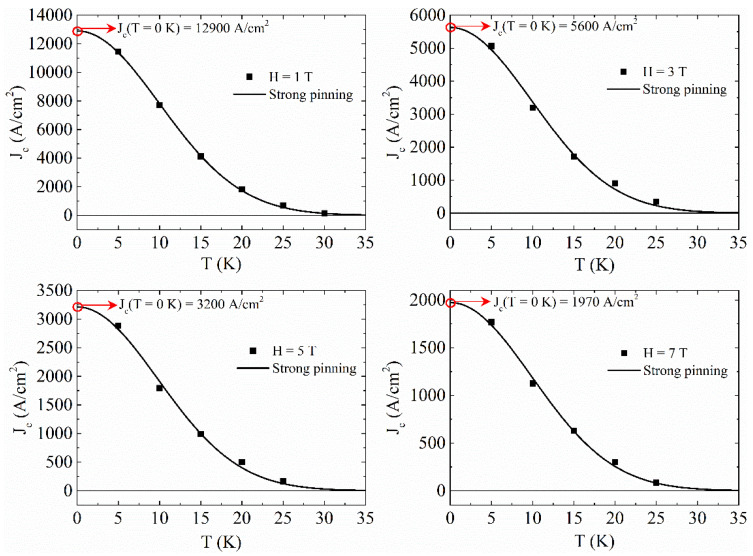
Temperature dependence of J_c_ at different applied magnetic fields fitted with strong pinning model (black solid line).

**Figure 6:**
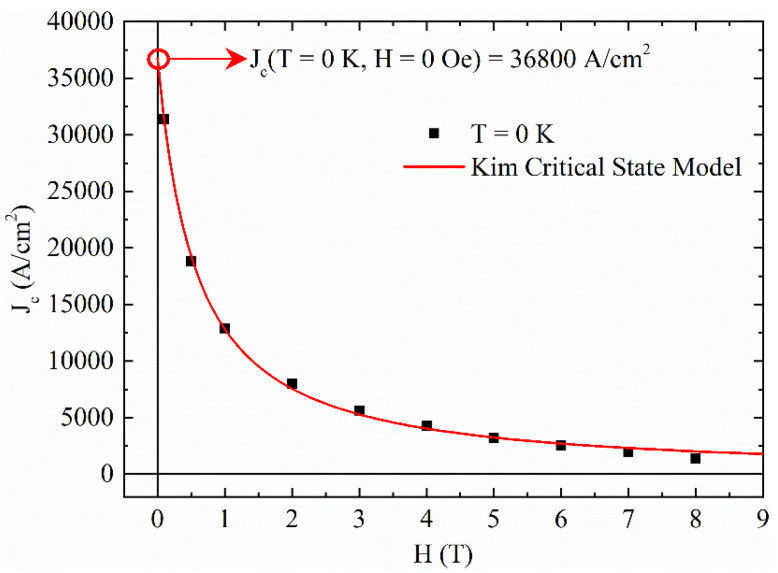
J_c_ at T = 0 K as a function of the magnetic field has been reported together with the fit with the Kim critical state model (red solid line).

Now, by fixing the field and by cutting the J_c_(H) obtained at different temperatures and reported in Figure 4, the J_c_(T) curves have been constructed and analyzed by using different equations reported in the literature describing different pinning models [34,35,36,37,38,39,40,41]. In all the field ranges, the best fit of the J_c_(T) curves has been obtained with equation considering the presence of strong pinning defects [42,43,44,45]

(2) $$J_{c}^{\mathrm{str}}\left(T\right)={\;J}_{c}^{\mathrm{str}}\left(0\right){\;e}^{-3{\left(T/T^{*}\right)}^{2}},$$

where $J_{c}^{\mathrm{str}}\left(T\right)$ is the temperature dependence of J_c_ in the framework of strong pinning regime, $J_{c}^{\mathrm{str}}\left(0\right)$ is J_c_ at T = 0 K and T* characterizes the vortex pinning by strong defect centers. The J_c_(T) curves fit for different magnetic fields is reported in Figure 5. It can be noted how the strong pinning model fits well our experimental data for all the considered field ranges. From these fits, the critical current density values at T = 0 K has been extracted as indicated with a red open circle in each of the panels of Figure 5, while T* ≈ 24 K for all the fits. Collecting these values, the J_c_(H) at T = 0 K has been obtained and reported in Figure 6. This curve has been fitted with several critical state models describing the field dependence of J_c_ [46,47,48,49,50,51]_._ The best fit has been obtained by using the Kim critical state model [47,48]

(3) $$J_{c}\left(H\right)=\frac{J_{c}\left(0\right)}{1+H/B_{k}},$$

where J_c_(0) is the value of J_c_ at H = 0 K and B_k_ is a parameter associated with the internal field. From the fit, B_k_ ≈ 0.63 T and the J_c_ at T = 0 K and H = 0 T, J_c_(0,0), can be obtained: J_c_(0,0) = 36,800 A/cm^2^.

**Figure 7:**
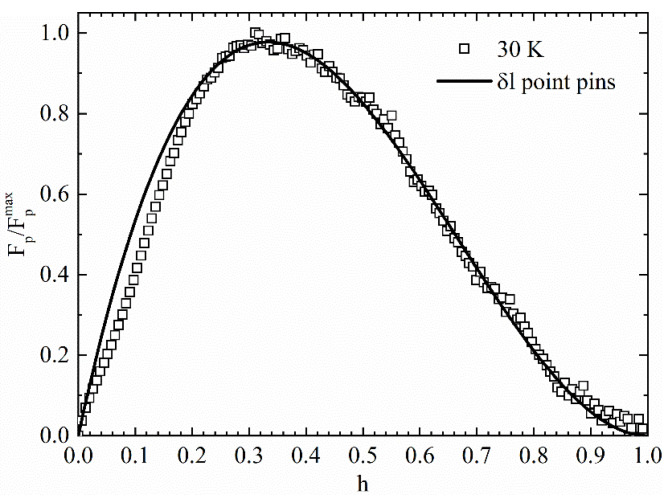
Normalized pinning force density as a function of the reduced magnetic field h = H/H_irr_ at T = 30 K fitted with Equation (4). Fit details are reported in the text.

The fit of the J_c_(T) curves by means of several pinning models is a powerful tool for discovering the pinning regime acting in the sample. On the other hand, it does not give specific information on the pinning defect type present in the sample. In this context, for deepening this aspect, it is helpful to study the behavior of the normalized pinning force density $F_{p}/F_{p}^{\max}$ as a function of the reduced magnetic field h = H/H_irr_ (where H_irr_ is the irreversibility field) by using the Dew–Hughes model [29]:

(4) $$\frac{F_{p}}{F_{p}^{\max}}={\mathrm{Ch}}^{p}{\left(1-h\right)}^{q},$$

where C is a proportionality constant, and p and q are fitting parameters that allow individuating the pinning defect type of the material. Equation (4) considers a maximum in the $F_{p}/F_{p}^{\max}$ vs. h behavior. In particular, for δl pinning the $F_{p}/F_{p}^{\max}$ maximum occurs at h_max_ = 0.33 with p = 1 and q = 2 in the case of point pins, at h_max_ = 0.20 with p = 0.5 and q = 2 in the case of surface pins, while no maximum occurs with p = 0 and q = 2 in the case of volume pinning [29]. For δT_c_ pinning, the maximum is expected for higher h than δl pinning [29]. In our case, H_irr_ = 6 T has been determined by taking the value of the magnetic field at which J_c_ ≈ 10 A/cm^2^ [52]. In Figure 7, the fit of $F_{p}/F_{p}^{\max}$ vs. h curve with Equation (4) has been reported.

The fit of Equation (4) with the experimental data gives h_max_ ≈ 0.33, p ≈ 1 and q ≈ 2, thus indicating that the point pins dominate the pinning mechanism inside our sample at T = 30 K. This has also been verified for other temperatures. It is worth to underline that this result is in agreement with the fit of the J_c_(H) at T = 0 K performed in Figure 6. In fact, the Kim critical state model can describe a superconductor having a homogeneous point defects distribution. In our opinion, since strong pinning regime has been found characterizing the sample, better current transport properties could be achieved with a better connection among the grains of our polycrystalline sample, which often is the key to enhancing the critical current density values in this class of superconductors [53,54,55,56,57].

## 4. Conclusions

We have studied the magnetic response of a CaKFe_4_As_4_ polycrystalline sample by using DC magnetization measurements as a function of the temperature and magnetic field. From the analysis of the Zero Field Cooling M(T) curve, T_c_ has been found equal to 35 K. A slight magnetic background has been found in the M(T) and M(H) curves for T > T_c_. In particular, due to the magnetic contribution, the superconducting hysteresis loops have shown a slight tilt, while the M(H) at 40 K has shown a coercive field different from zero, suggesting the presence of a ferro/ferrimagnetic phase or of magnetic impurities inside the sample. However, the magnetic contribution did not affect the critical current density calculated starting from the M(H) curves. In particular, the field dependence of the critical current density J_c_(H) has shown values in agreement with other polycrystalline samples present in the literature. By analyzing the J_c_(T) curves at different magnetic fields and the F_p_(h), it has been found that a strong pinning regime characterized by point pins acts in the sample for all the field ranges. Finally, the J_c_(H) at T = 0 K has been obtained and fitted in the framework of the Kim critical state model coherently with the presence of point pins in the sample. Although the current transport capabilities are not excellent, the obtained results suggest that the enhancement of the number of the strong defects and a better connection among grains, which can be obtained by manipulating the fabrication processes, can significantly increase the critical current density values of the sample.

## Data Availability

The data sets that support the findings in this study are available from the corresponding author upon reasonable request.
